# Isolation and Characterisation of the Agarolytic Bacterium *Pseudoalteromonas Ruthenica*

**DOI:** 10.1515/biol-2019-0066

**Published:** 2019-12-31

**Authors:** Ashraf Khalifa, Munira Aldayel

**Affiliations:** 1 King Faisal University, Hofuf Saudi Arabia

**Keywords:** Agarolytic bacteria, BIOLOG, Halophilic bacteria, *Pseudoalteromonas*

## Abstract

Agar is a polysaccharide that primarily constitutes the cell wall of red algae. It is a good source of carbon and energy for many microbes. In the present study, an agarolytic bacterium, UQAD-3, was obtained from the waters of Al-Uqair, the Arabian Gulf, Al-Ahsaa, Saudi Arabia. UQAD-3 exhibited agarolytic activity when grown on agar as the sole source of carbon and energy. The strain was identified as *Pseudoalteromonas ruthenica* based on comparative analysis of the 16S rRNA, with 99.6% similarity. This finding was further confirmed by phylogenetic analyses based on 16S rRNA gene sequences, which highlighted that UQAD-3 was assembled within the Pseudoalteromonas clade and constituted a monophyletic subcluster with P. ruthenica, KMM 300^T^. The strain was further characterised biochemically using the Biolog Gen III microtest system. UQAD-3 showed positive reactions to 16 (17%) of the 94 diverse traits assessed. Good growth was reported in 10% NaCl indicating its moderate halophilic nature. These observations indicate the agarolytic potential of the strain and opens new horizons for industrial applications in the future.

## 1 Introduction

Marine bacteria are considered as a valuable source for important metabolites that can be exploited in various biotechnological applications. Marine environments present harsh conditions such as high salinity, high pressure, and low temperature. Therefore bacteria inhabiting such environments harbour ecological and physiological advantage over other bacterial species.

The enzymatic machinery of bacteria in such habitats produce enzymes and unique metabolites such as vitamins, drugs, biosurfactant molecules and many other bioactive compounds with unpreceded applications in industry and medicine [1, 2, 3]. For instance, hydrolases play an important role in the textile, food, pharmaceutical, and paper industries. Production of α-amylases from *Chromohalobacter* sp. [4] and actinomycin X2 and fungichromin from *Streptomyces padanus* [5] are a few examples.

Al Uqair is on the western coast of the Arabian Gulf and is about 50 miles southeast of Al-Ahsaa oasis, Eastern province, Saudi Arabia. The percentage of total soluble salts in Al Uqair water is greater than 18%.

Seaweeds have large quantities (~60% of the dry weight) of agar in their cell wall [6]. Agar is a heterogeneous polysaccharide that is mainly made up of agarose and agaropectin. Various microbes metabolise agar as their only source of carbon and energy using agarases. Agarolytic bacteria include *Pseudoalteromonas* sp. associated with three antarctic subtidal macroalgae [7], *Ammoniibacillus agariperforans* obtained from compost [8], and *Flammeovirga* sp. solated from coastal sediments [9]. These microbes inhabit various ecological niches.

Breakdown of agar by agarases releases high-value compounds that have various properties such as anti-inflammatory, antibacterial, and antioxidant activities [10, 11]. Additionally, agarases have a pivotal role in carbon cycling as they accomplish the key steps in agar degradation, highlighting the environmental role of agarases. They also play an important role in the generation of protoplasts (living plants cells devoid of cell walls) [12]. Protoplasts provide an appropriate single cell system that facilitate several biotechnological experiments such as genetic transformation and metabolic engineering [12]. Furthermore, agarases are useful for investigating the composition and structure of cell wall polysaccharides of seaweeds. Additionally, agarases facilitate in recovering DNA from agarose gel [13].

Despite the aforementioned importance of agarases, agar-degrading bacteria in Al-Ahsaa is largely overlooked. However, agarolytic bacteria, *Halomonas aquamarina* and *Alteromonas macleodii* were obtained from the Arabian Gulf coast in Kuwait City. Such strains have been reported to metabolize hydrocarbons and to fix nitrogen non-symbiotically [14]. Therefore, it was of interest to explore other sites of the Arabian Gulf for agarolytic bacteria particularly in the Al-Uqair coast, Al-Ahsaa, Saudi Arabia. To achieve this, water samples were collected from Al-Uqair. The isolated strain was phenotypically and biochemically characterised and genetically identified.

## 2 Materials and methods

### 2.1 Isolation of agarolytic strain UQAD-3 from Al Uqair

Water samples were collected in sterilized plastic bottles (250 mL) from Al Uqair 25° 38′ 35″ N, 50° 12′ 52″ E, Eastern region, Saudi Arabia. The water samples were sent to the laboratory wherein enrichment with agar (0.5% w/v) as the only source of carbon and energy was carried out in a 250 mL conical flask containing 50 mL of Nitrate Mineral Salts (NMS) liquid medium [15]. Incubation was carried out at 28°C in a shaking-incubator at 150 rpm m^-1^ for 7 days. Subsequently, aliquots (100 μL) from the agar-enriched medium were streaked onto NMS agar plates and were incubated at 28°C for 7 days. Single colonies that formed an agar depression and a clear halo upon addition of a few drops of iodine, were picked and streaked onto fresh NMS agar plates. The strain that produced the highest agarase activity based on the halo zone was selected for further study. The strain was designated UQAD-3 and was maintained by regular streaking on NMS plates every 2–3 weeks.

### 2.2 Qualitative assay for agarolytic activity of the strain UQAD-3

The ability of the strain UQAD-3 to degrade agar was further verified qualitatively. A loopful of the strain UQAD-3 was carefully spotted on fresh NMS agar plates. Appearance of a clear zone around the colonies upon addition of Logol’s iodine indicated agarolytic activity.

### 2.3 Determination of agarase activity using Dinitrosalicylic acid (DNSA)

Quantitative agarase activity of the strain UQAD-3 was determined using the 3,5‐dinitrosalicylic acid (DNSA) method described earlier [16]. This method measures the amount of reducing sugars (galactose) released. Briefly, 50 μL enzyme was added to a 950 μL Tris/HCl buffer (20 mM, pH 8, buffer A) solution containing 0.2% (w/v) agarose as the substrate. The reaction mixture was incubated at 30 °C for 30 min. The sample was mixed with 500 μL of 3,5-dinitrosalicylic acid reagent solution (0.65 g of DNS, 4.5 mL of glycerol, and 32.5 mL of 2 N NaOH in 100 mL of distilled water). 50 μL water was used as the negative control. The tubes were heated in boiling water for 10 min, and subsequently cooled in an ice bath. OD values were measured at 540 nm. Standard curve was obtained using d‐galactose as the standard. One unit (U) of enzymatic activity was defined as the amount of enzyme that released 1 μmol of reducing sugar per minute under this condition.

### 2.4 Production and separation of agarase enzyme

For production of agarase, the strain UQAD-3 was grown in 250 mL NMS medium supplemented with 0.3 % (w/v) agarose as the sole carbon source at 28 °C for 48 h. Next, 25 mL of the actively growing UQAD-3 strain was measured accurately and was inoculated in a 1000 mL Erlenmeyer flask containing 250 mL of NMS medium supplemented with 0.3% agar as the sole carbon and energy source. The flasks were incubated at 28°C for 72h at 150 rpm. After the incubation, cells were precipitated by centrifugation at 12000 × g for 20 minutes at 4°C. The supernatant served as the crude for agarase enzyme. Recovery of the enzymes was performed by salting out process following the method described previously [17].

### 2.5 SDS-PAGE analysis

The enzyme fractions were run on an SDS-PAGE gel using a 11% separating gel (pH 8.8) and a 5% stacking gel (pH 6.8). The resolved proteins were detected by Coomassie brilliant blue staining against protein molecular weight markers by Thermo Scientific™ PageRuler™ Plus Prestained 10-250 kDa Protein Ladder.

### 2.6 External features of the strain UQAD-3

The colonial morphology of the strain UQAD-3 was determined for 3-day old colonies growing on NMS agar plates.

### 2.7 Determination of cell shape using scanning electron microscopy

Cell shape were determined using SEM as previously described [18].

### 2.8 Biochemical characterisation using Biolog Gen III microtest system

The biochemical characteristics of the strain UQAD-3 were investigated using the Biolog Gen III microtest system (Biolog, USA) following the instructions of the manufacturer. The 96-well plate was incubated at 28°C for 24 h. Next, the responses of the strain were reported visually based on the colour change. It is worth mentioning that the plate included appropriate negative and positive controls where colourless and purple colour were observed, respectively.

### 2.9 Salinity tolerance

In order to determine the extent of salinity tolerance, a loopful of 48 h-old UQAD-3 culture that was grown on nutrient agar plates at 28°C, were spread on nutrient agar plates supplemented with different NaCl concentrations (0, 1, 2, 3, 4, 5, 6, 7, 8, 9, 10, and 15% NaCl). Plates were incubated at 28°C for 3 days. After incubation, the growth was reported visually. The experiment was performed in triplicates.

### 2.10 Extraction of genomic DNA

DNA was extracted from a single colony of the UQAD-3 strain using the InstaGene Matrix Kit (Bio-Rad, Hercules, CA, USA), according to the instructions of the manufacturer.

### 2.11 PCR amplification of 16S rRNA gene

Amplification of the 16S rRNA gene was performed using the universal primers, 27f/1492r with the conditions described in [19]. PCR products were purified and sequencing of the 16S rRNA gene was carried out as previously mentioned [20] .

### 2.12 Phylogenetic analyses

Phylogenetic relationships between the strain UQAD-3 and its closely related recognized bacterial species were inferred from a neighbour-joining tree. The tree was constructed according to the Tamura-Nei model [21] including all the codon positions using the MEGA 7 [22]. To determine the branch support, 1,000 bootstrap replicates were analysed. The 16S rRNA gene sequence obtained for the strain UQAD-3 has been deposited in the NCBI GenBank (accession number MF288900).

## 3 Results and discussion

In the current study, we isolated three-agarolytic bacterial isolates, namely, UQAD-1, UQAD-2, and UQAD-3, from the western coast (Al Uqair) of the Arabian Gulf. The morphological characteristics are presented in Table 1. UQAD-1, UQAD-2, and UQAD-3 formed white, yellow, and pale orange colonies respectively. However, they exhibited similar shape, elevation, and margin. UQAD-3 was selected based on the qualitative test for the agarolytic activity for further phenotypic and genotypic characterisation. Enrichment with agar as the sole source of carbon and energy was proven effective in obtaining the strain UQAD-3.

**Table 1:** Morphological characteristics of the colonies formed by the bacterial strains

Feature	UQAD-1	UQAD-2	UQAD-3
Colour	White	Pale yellow	Pale orange
Shape	Circular	Circular	Circular
Elevation	Convex	Convex	Convex
Margin	Entire	Entire	Entire
Diameter of halo zone after addition of iodine	3 ± 0.20	4 ±0.25	7 ± 0.42

### 3.1 Phenotypic characterization of the strain UQAD-3

**Figure 1:**
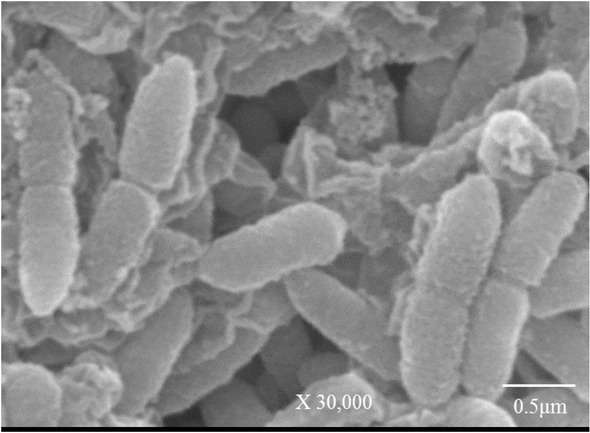
Scanning electron micrographs showing the cell shape and arrangement of the strain UQAD-3. Magnification and scale bar are shown at the bottom of the image.

UQAD-3 formed circular colonies with a complete margin. The colonies produced a pale orange colour (Table 1). As shown in Figure 1, the UQAD-3 cells were rod-shaped, single or in pairs.

**Figure 2:**
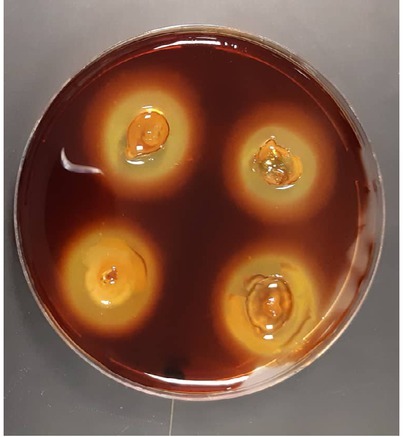
Agar degradation of the strain UQAD-3. A clear halo zone was formed around the colony upon addition of drops of iodine solution.

The agarolytic potential was verified via appearance of depression on agar underneath the growing strain, formation of a clear zone after addition of a few drops of iodine (Figure 2), and liquefaction of NMS medium containing 0.5% agar as the sole source of carbon and energy. Additionally, agarase activity was determined

for the actively growing UQAD-3 as 0.44 units mL^-1^ (Table 2). This value is lower than the agarase activity of *Pseudoalteromonas* CY24, which was 17 units mL^-1^ [23]. Therefore, optimisation of the conditions for agarase activity is required in the future. These findings indicate the robustness of the agarolytic nature of the strain UQAD-3. A group of hydrolytic enzymes named agarases mediates the first step in the agar degradation pathway. Agarases vary in their pattern of agar cleavage into α-agarase, β-agarase, and β-porphyranase [24]. Whether the strain UQAD-3 produces α-agarase or β-agarase, requires further study.

**Table 2:** Characterisation of the strain UQAD-3

Feature	UQAD-3
**Physiological and biochemical characteristics (Biolog Gen III MicroPlate test system)**	
α-D-Glucose	+
D-Fructose	+
D-Gluconic Acid	+
L-Lactic Acid	+
D-Cellobiose	+
D-Salicin	+
Glycerol	+
Acetoacetic Acid	+
1% NaCl	+
1% Sodium Lactate	+
Nalidixic Acid	+
Lithium Chloride	+
Sodium Butyrate	+
8% NaCl	+
pH 4	+
Potassium Tellurite	+
D-Glucuronic Acid	/
Glucuronamide	/
Tetrazolium Violet	/
Growth at 10% NaCl	+
Growth at 15%	-
GenBank with an accession number (MF288900)	
Agarase activity using DNSA	0.44 unit mL^−1^

*: - negative reaction; **: + positive reaction; ***: / borderline.

**Figure 3:**
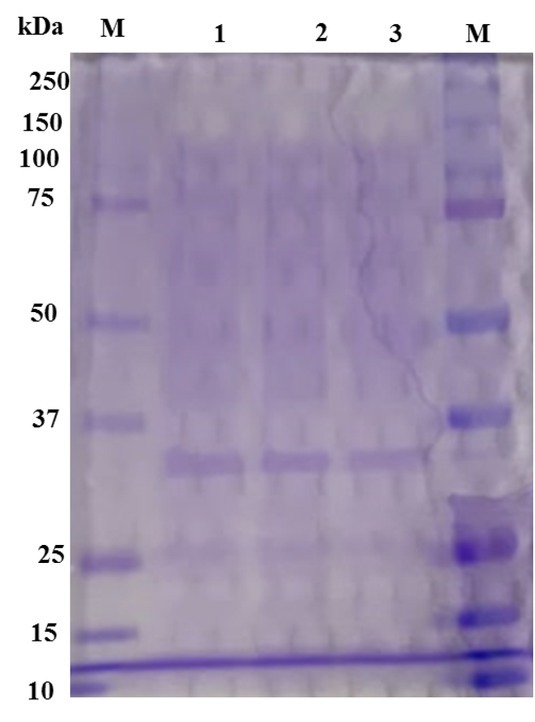
SDS-PAGE analysis of agarase. Lane M PageRuler™ Prestained Protein Ladder; lanes 1, 2 and 3 agarase.

Precipitation of protein in the supernatant was carried out using ammonium sulphate. SDS-PAGE showed a single concrete band with an average weight of 30 kDa (Figure 3). This weight is slightly lower than the molecular weight of agarase in *P. antarctica* N-1 (33 kDa) [25]. In addition, the agarase reported in this study is likely to be classified within group I (20–49 kDa) of β-agarases based on the molecular weight [26]. It is worth mentioning that agarases vary considerably with respect to their molecular weights; from as low as 12 kDa in *Bacillus megaterium* [27] to as high as 210 kDa in *Pseudomonas*-like bacteria [28].

The commercially available microtest system, Biolog Gen III, was used as a fast and effective tool for further biochemical characterisation of UQAD-3 (Table 2). UQAD-3 reacted positively to 16 (17%), of the 94 diverse traits examined (Table 2). (UQAD-3 grew in the presence of either α-d-glucose, d-fructose, d-gluconic acid, l-lactic acid, d-cellobiose d-salicin glycerol, or acetoacetic acid; this highlights the fact that UQAD-3 has metabolic variability in consuming various metabolites as the sole source of energy and carbon, in addition to its ability to degrade agar using agarase. For instance, the utilisation of d-cellobiose is attributed to the hydrolytic enzyme (cellobiose phosphorylase or β-glucosidase) that can release free glucose units that can be further metabolised by the strain for energy [29]. Similar results have recently been reported, which show that the agar-degrading marine bacterium *Marinomonas agarivorans* could utilise a range of carbohydrate compounds as a single source of carbon and energy [30]. The metabolic diversity may play a pivotal role in the survival and competition of the strain in its natural ecosystem).

Additionally, UQAD-3 grew well in the presence of 10% NaCl but no growth was observed at 15% NaCl indicating the moderate halophilic nature of the strain.

These findings were expected as the strain was originally obtained from a saline marine ecosystem. Bacteria that inhabit saline environments use various mechanisms to adapt to the elevated salt concentration. One common mechanism is to maintain the internal ion concentration at a level that allows normal metabolic activity with the least possible damage [31]. This can be achieved via upregulation of ion exporters. Additionally, production of osmoregulators and osmoprotectants are also important mechanisms to control the adverse effects of salinity stress [31]. Such halophilic bacterial strains could be exploited biotechnologically. Economically important transgenic plants with salt-tolerance features could be obtained via appropriate genetic engineering tools using the bacterial strains as the source of the genes conferring this trait.

### 3.2 Identification of bacterial strain UQAD-3 using the 16S rRNA sequencing

**Figure 4:**
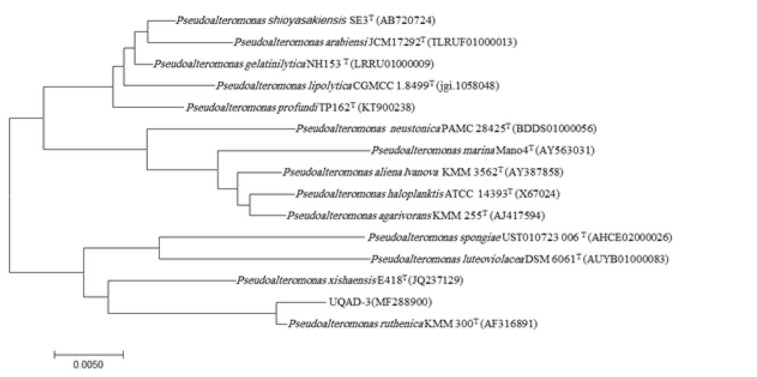
Evolutionary relationships of the strain UQAD-3 and closely related reference species. The evolutionary history was inferred using the neighbour-joining method [32]. The optimal tree with branch length sum = 0.14946950 is shown. The tree is drawn to scale, with branch lengths in the same units as those of the evolutionary distances used to infer the phylogenetic tree. The evolutionary distances were computed using the Maximum Composite Likelihood method [34] and are in the units of the number of base substitutions per site. The analysis involved 17 nucleotide sequences. Codon positions included were 1st+2nd+3rd+Noncoding. All positions containing gaps and missing data were eliminated. There were a total of 1352 positions in the final dataset. Evolutionary analyses were conducted in MEGA7 [22].

Comparative analysis of the 16S rRNA gene identified the strain as *P. ruthenica*, with 99.6% similarity. This finding was further confirmed by the phylogenetic analyses based on 16S rRNA gene sequences which highlighted that UQAD-3 was assembled within the *Pseudoalteromonas* clade and constituted a monophyletic subcluster with *P. ruthenica*, KMM 300^T^ (Figure 4). The evolutionary history tree was inferred using the neighbour-joining method [32] using MEGA7 [22]. Sequencing of the 16S rRNA gene has been used as the gold standard method of bacterial identification at the genus and species levels as well as for inferring phylogenetic relationships among prokaryotic organisms [33].

## 4 Conclusions

In this study, an agarolytic and halophilic bacterial strain UQAD-3, was isolated from a marine ecosystem in the Al Ahsaa region of Saudi Arabia and identified as *P. ruthenica* based on comparative 16S rRNA analysis. The strain was further characterised biochemically using the Biolog Gen III microtest system. The agarolytic activity of this strain was attributed to agarases. Agarases are multipurpose enzymes that play important roles in carbon cycling, recovery of DNA from agarose gels, and production of high-value compounds that have various activities such as anti-inflammatory, antibacterial, and antioxidant activities. Recovery of agarases from a local strain in the Al Ahsaa region could open new horizons for industrial applications in the future.
